# Transcriptome and Metabolome Profiling Unveil Pigment Formation Variations in Brown Cotton Lines (*Gossypium hirsutum* L.)

**DOI:** 10.3390/ijms24065249

**Published:** 2023-03-09

**Authors:** Yin-Ping Lv, Gang Zhao, Yong-Fei Xie, Anane Gideon Owusu, Yong Wu, Jun-Shan Gao

**Affiliations:** School of Life Sciences, Anhui Agricultural University, Hefei 230036, China

**Keywords:** cotton fiber, flavonoid biosynthesis, anthocyanin biosynthesis, transcriptomics, metabolomics

## Abstract

Naturally brown colored cotton (NBCC) is becoming increasingly popular due to its natural properties of coloration. However, poor fiber quality and color fading are key issues that are hindering the cultivation of naturally colored cotton. In this study, based on transcriptome and metabolome of 18 days post-anthesis (DPA), we compared the variations of pigment formation in two brown cotton fibers (DCF and LCF), with white cotton fiber (WCF) belonging to a near-isogenic line. A transcriptome study revealed a total of 15,785 differentially expressed genes significantly enriched in the flavonoid biosynthesis pathway. Furthermore, for flavonoid biosynthesis-related genes, such as flavonoid 3′5′-hydroxylase (*F3′5′H*), anthocyanidin synthase (*ANS*), anthocyanidin reductase (*ANR*), chalcone synthase (*CHS*), dihydroflavonol 4-reductase (*DFR*), and chalcone isomerase (*CHI*), their expressions significantly increased in LCF compared with DCF and WCF. Moreover, transcription factors *MYB* and *bHLH* were significantly expressed in LCF and DCF. Most flavonoid-related metabolites (myricetin naringenin, catechin, epicatechin-epiafzelechin, and epigallocatechin) were found to be more highly up-regulated in LCF and DCF than WCF. These findings reveal the regulatory mechanism controlling different brown pigmentation in cotton fibers and elucidate the need for the proper selection of high-quality brown cotton fiber breeding lines for promising fiber quality and durable brown color pigmentation.

## 1. Introduction

Cotton’s raw materials, especially its fiber, are widely used on a daily basis worldwide. Cotton is an essential natural fiber and is one of the world’s leading fiber crops, being grown in more than 80 countries [1]. Naturally colored cotton fibers have natural coloration, which provide fibers with colors other than white [2]. Both colored and white cotton fiber are ancient cotton types that have been grown and used by mankind since 2500 B.C. in India, China, and central Asia [3]. Primarily, two naturally colored cottons (brown and green) have been extensively studied [4]. However, recent cross-breeding of both brown and green cotton fiber color has led to several shades of different cotton colors. These cotton types are lucrative components for textile industries due to the necessity of not dying cotton during the processing and manufacturing of the fabric, as it creates no hazardous dye waste, saves manufacturing costs, and is more ecologically friendly [5,6]. 

Naturally brown colored cotton (NBCC) is the most common type of cotton found in different shades. Depending on the intensity of color, NBCC can be called light brown, camel color, deep brown, dirty grey, tan, or red [3]. Apart from the common features of NBCC, when compared to that of the conventional white counterparts, NBCC fibers have been found to contain excellent antibacterial properties, antioxidant properties, and durable scouring and laundering [7]. Despite these excellent differences, other investigations have also proven that naturally colored cotton’s fiber color is monotonous and unstable, which limits its use and manufacturing [2,8]. Although naturally colored cotton is known to be unstable, NBBCs were reported to be more stable than green colored fiber (GCF) [3]. GCF pigmentation structure is thought to be complex, however, it is also chemically suberized and waxy, and is controlled by the phenylpropanoid biosynthesis pathway with higher flavonoid components compared white colored fiber (WCF) [9,10]. Contrary to brown cotton fiber (BCF), the composition of pigment structure and related genes expressed resulted in proanthocyanidins (PAs) being the main pigment [11]. PAs and their derivatives are mainly generated from the flavonoid pathway characterized by several genes, encoding *CHS*, *F3H*, *DFR*, *F3′5′H*, *LAR*, *ANS*, and *ANR* [8,12]. The structural content of PAs has been reported in both white and brown fibers. However, Mikhailova et al. further explained that prodelphinidin and PA contents are equal in white cotton, but in brown, 90% of prodelphinidin in comparison to 10% PA was found [13]. Chemically, PAs are colorless polymers that are synthesized by adding flavan-3, 4-diol molecules to an initiating flavan-3-ol unit via the flavonoid pathway [11,14]. The flavan-3-ol unit monomers formation pathway varies, with tissues and species determined by PA compositions in different plants. Nonetheless, in brown cotton fibers, these PA units (gallocatechin and catechin) have been confirmed to accumulate significantly [11,15]. 

As explained above, the brown color is found in different shades, ranging from light brown to intense mahogany red. Physiologically, brown cotton colors are often mistaken to have a more stable color pigmentation compared to other colors depending on the color intensity, but with continuous exposure to sunlight, the brown color gradually fades at a very slow rate. This shows that mere physical observation of a deep color in brown cotton fibers might not guarantee pigment stability. The molecular study of brown colors, including respective genes and pathway analysis, is extensively understood [4,5,16,17]. However, there is less existing information regarding pathway differences, genes expression, and potential similarities among NBCCs. Therefore, in this study, we compared the deep brown cotton fiber (DCF), WCF, and light brown cotton fiber (LCF) of *Gossypium hirsutum L*. using transcriptomics and metabolomics tools at 18 DPA. Our means to attain this goal was to first classify significant differential metabolites during DCF, WCF, and LCF color development using metabolomics analysis. Subsequently, structural genes involving flavonoid pathway were obtained through analysis of differentially expressed genes. Our results can provide a systems-level context for advanced studies on the gene regulation network of anthocyanin biosynthesis pathways involved in NBCC and can contribute to the breeding of light brown cotton cultivars.

## 2. Results

### 2.1. Overview of Metabolomics Profiling 

**Figure 1:**
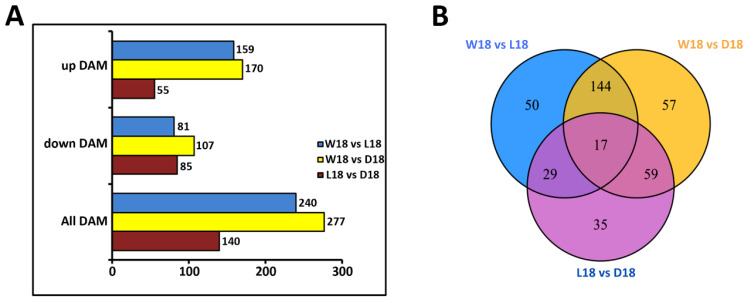
Differentially expressed metabolites analysis. (**A**) Bar chart of metabolite expression levels in D18, L18, and W18. (**B**) Venn diagram showing different metabolites identified between WCF, LCF, and DCF. L18, D18, and W18 represent deep brown fiber, light brown fiber, and white fiber at 18 DPA.

To evaluate the metabolite differences in WCF, LCF, and DCF, datasets obtained from LC−MS/MS were subjected to principal component analysis (PCA). The number of differentially expressed metabolites (DEM) and pairwise comparison of expressed metabolites detected were conducted (W18 vs. L18; W18 vs. D18; L18 vs. D18). Based on a fold-change threshold >1.5, we identified 240 DEM (159 up-regulated; 81 down-regulated) in W18 vs. L18, 277 DEM (170 up-regulated; 107 down-regulated) in W18 vs. D18, and 140 DEM (55 up-regulated; 85 down-regulated) (Figure 1A). Generally, more up-regulated metabolites and fewer down-regulated metabolites were observed. Out of these metabolites, 391 DEM were assigned for Venn analysis and 17 were found to be commonly different for all three cotton fiber colors (Figure 1B). 

### 2.2. Untargeted Metabolome Analysis of Fiber Coloration in Cotton at 18 DPA

**Figure 2:**
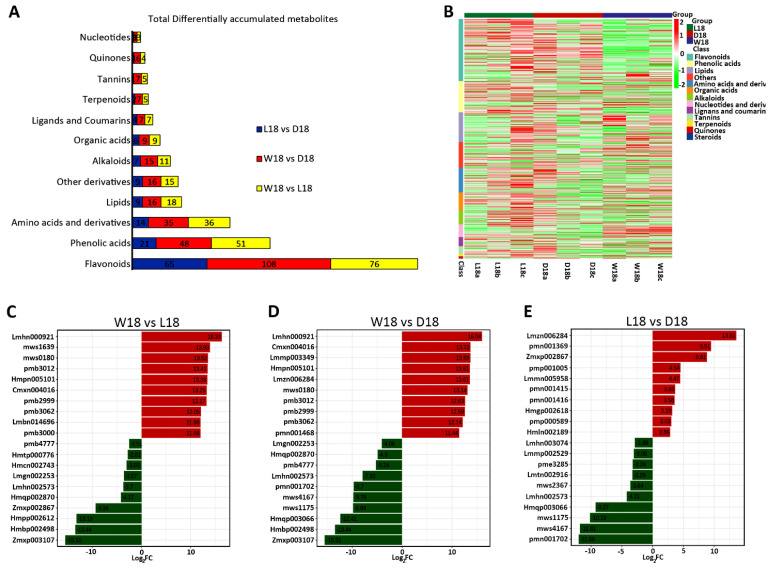
Classification of differentially expressed metabolite. (**A**) Total number differentially accumulated metabolites classes. (**B**) A heat map classification of metabolite groups. Horizontal represent cotton types at 18DPA. Vertical represent differentially expressed metabolite classes. (**C**) Top 20 DEMs in W18 versus L18. (**D**) Top 20 DEMs in W18 versus D18. (**E**) Top 20 DEMs in L18 versus D18. The abscissa is the log_2_FC of the differential metabolite and the ordinate is the differential metabolite. The red color represents up-regulation of DEMs and green represents down-regulation of DEMs.

**Figure 3:**
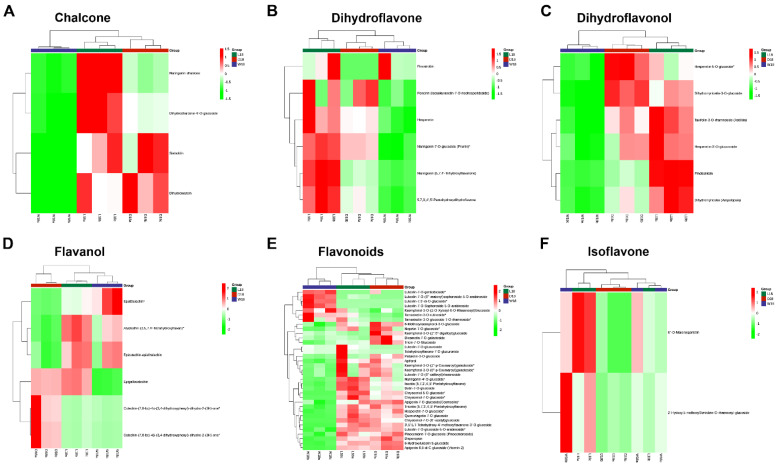
Clustering analysis of DEMs. (**A**) Chalcone DEMs. (**B**) Dihydroflavone DEMs. (**C**) Dihydroflavonol DEMs. (**D**) Flavonol DEMs. (**E**) Flavonoids DEMs. (**F**) Isoflavone DEMs. Red color represents up-regulation of differentially expressed metabolites and yellow represents down-regulation of differentially expressed metabolites. L18, D18, and W18 represent deep brown fiber, light brown fiber, and white fiber at 18 DPA.

Through Kyoto encyclopedia genes and genomes (KEGG) pathway annotation of metabolites, varieties of functional pathways were discovered. However, DEMs highly expressed in major metabolic pathways in all three comparisons (W18 vs. L18; W18 vs. D18; L18 vs. D18) were metabolic pathways, biosynthesis of secondary metabolites, flavonoid biosynthetic pathway, phenylpropanoid biosynthetic pathway and flavone, and flavonol biosynthetic pathway (Figure S1). As shown in Figure 2A, 13 DEMs classified groups were significantly expressed, including amino acids and derivatives (89), phenolic acids (114), nucleotides and derivatives (46), flavonoids (226), quinones (7), lignans and coumarins (33), tannins (29), alkaloids (51), terpenoids (9), steroids (1), lipids (109), organic acids (66), and others (93). Flavonoids pigmented metabolites were found to be highly accumulated (Figure 2A). A heat map study revealed a metabolite class with a high number of DEMs in all three cotton fibers colors. LCF showed a high accumulation of flavonoids compared to WCF and DCF (Figure 2B). According to top 20 DEMs, in WCF and LCF, the highly significantly up-regulated DEMs were tannin compound (lmhn000921), coumarins compound (mws1639), phenolic acids (mws0180 and pmb3062), flavonoid compounds (pmb3012 and pmb2999), and chalcone compounds (hmpn005101 and cmxn004016), while the highly significant down-regulated DEMs were flavonoid compounds (zmxp003107, hmpp002612, and zmxp002867) and coumarin compound (hmbp002498) (Figure 2C and Table S1). Similarly, in WCF and DCF, tannin metabolite compound (lmhn000921), chalcone compounds (hmpn005101 and cmxn004016), other compounds (lmmp003349), triterpene (lmzn006284), phenolic acid (mws0180), and flavonoid compound (pmb3012) were highly up-regulated DEMs. The highly significant down-regulated DEMs were flavonoid compounds (zmxp003107, mws4167, pmn001702, mws1175, and hmqp003066) and coumarin compound (hmbp002498) (Figure 2D and Table S2). Lastly, in LCF and DCF combination, triterpene compound (lmzn006284), lignin compound (pmn001369), and flavonoid compound (zmxp002867) were extremely expressed DEMs. Contrarily, significant down-regulated DEMs were flavonoid compounds (pmn001702, mws4167, mws1175, and hmqp003066) (Figure 2E and Table S3). Interestingly, the DEMs associated with pigment adjustment, such as flavonoids [18], chalcones [4], and flavanols, were found to accumulate significantly in W18_vs_L18 and W18_vs_D18. In addition, the DEMs in W18 vs. D18, and W18 vs. L18, related to flavonoids biosynthesis, had high accumulation. This confirms that flavonoids could induce the changes of color in cotton fiber. Clustering analysis revealed the expression rates of different related metabolites in DCF, WCF, and LCF. Highly up-regulated metabolites were found in LCF compared to DCF and more down-regulated metabolites were found in WCF. For example, the majority of chalcone, dihydroflavone, dihydroflavonol, flavanol, flavonoid, and isoflavones were highly expressed in LCF to DCF and WCF, with few exceptions (Figure 3A–F).

### 2.3. De Novo Transcriptome Assembly and Gene Expression Profiles in WCF, LCF and DCF at 18 DPA

With the aim of logically identifying of the significant genes affecting the cotton fiber color development, we performed transcriptome sequencing after 18 DPA in white cotton fiber (S18), brown cotton fiber (B18), and light brown cotton fiber (Q18). Together, 9 RNA-seq libraries of fiber samples were constructed and analyzed at 18 DPA. A total of 71.26 Gb of clean data were obtained after removing of low-quality reads (Table S4). The proportion of clean data that were mapped to the genome of *G. hirsutum* reached a total of 91.30% to 92.28%, with no less than 89.02% of the Q30 and over 44.17% of the GC content, signifying a reliable quality of the RNA-seq result.

**Figure 4:**
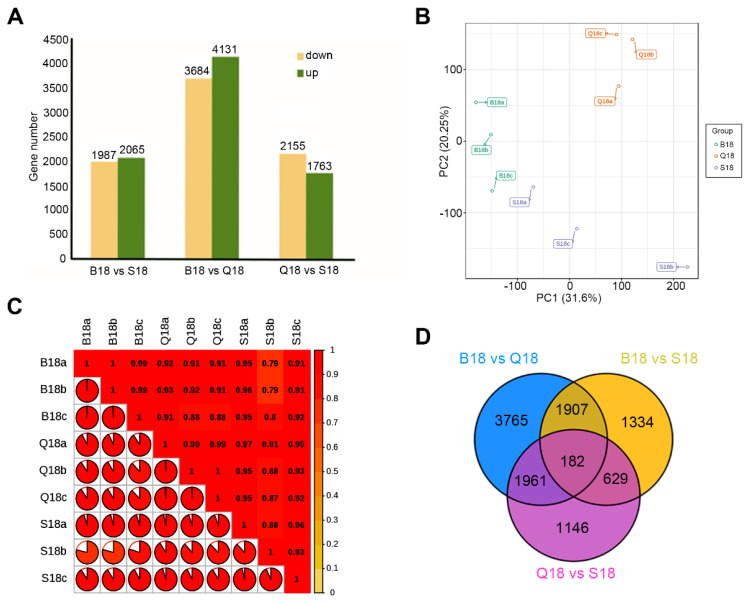
Identification of DEGs between DCF, LCF, and WCF. (**A**) The total number of DEG identified in each comparison. (**B**) Principal Component Analysis of the genes identified from the 9 samples. (**C**) Pearson Correlation Coefficient of the genes identified from the 9 samples. (**D**) Venn diagram showing DEGs between DCF, LCF, and WCF. B18, Q18, and S18 represent DCF, LCF, and WCF at 18DPA, respectively.

A total of 15,785 differentially expressed genes (DEGs) between B18, S18, and Q18 at 18 DPA were found. As shown in Figure 4A, among the three combinations, up-regulated gene numbers were higher than down-regulated gene numbers. However, B18 vs. Q18 (4131 up-regulated; 3684 down-regulated) had the highest number of DEGs. For further understanding of the relationship between the 9 RNA-seq libraries, principal component analysis (PCA) was performed (Figure 4A). The results showed that genes from different cotton colors (B18, S18, and Q18) at 18 DPA were clearly separated in the score plots. In addition, the first (PC1) and second (PC2) principal component represented 31.60% and 20.25% of the total variations, respectively (Figure 4B). The PCA analysis indirectly demonstrates, to some extent, the consistency of our RNA-seq data. Moreover, Pearson correlation coefficient analysis showed high correlations between compared samples in B18 and Q18 compared to S18 at a coefficient rate of R^2^ = 0.8. This hinted at significant gene expression alteration at the stage of BCF and LCF cotton fiber color development (Figure 4C). Furthermore, 15,785 DEGs were assigned for Venn analysis at 18 DPA. A total of 7815 DEGs were uniquely found by comparing B18 versus Q18, 4052 DEGs by comparing B18 versus S18, and 3918 DEGs by comparing Q18 versus S18 (Figure 4D). Lastly, 182 were differentially expressed at all the three combinations. The number of differential expressed genes between B18 and Q18 increased was significantly higher compared to other combinations (Figure 4D).

### 2.4. KEGG Pathway Enrichment and GO Functional Classification Analysis of DEGs

**Figure 5:**
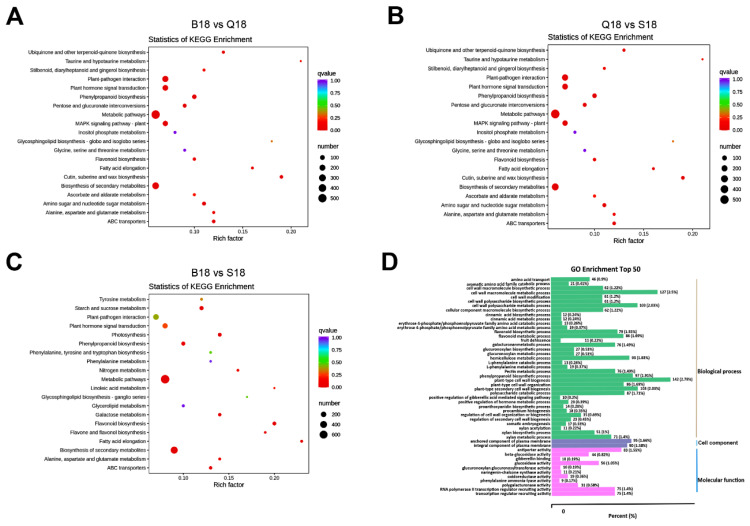
Results of top 20 KEGG and 50 GO enriched pathways analysis at 18 DPA. (**A**) KEGG enriched pathways between DCF and LCF. (**B**) KEGG enriched pathways between LCF and WCF. (**C**) KEGG enriched pathways between DCF and WCF. Q-value is a *p*-value that has been adjusted for the false discovery rate (FDR). The lower q-value indicates that a lower percentage of significant results will be false positives. Rich factor represents the degree of enrichment of genes under the designated pathway term. The greater the rich factor value, the greater the degree of pathway enrichment. (**D**) Fifty GO enriched pathways analysis. Green represents enrichment in biological process, purple represents enrichment in cell components, and pink represents enrichment in molecular function.

In order to significantly classify induced pathways in cotton fiber colors at 18 DPA, all DEGs were subject to KEGG pathway enrichment analysis. The DEGs were enriched to 130 KEGG pathways in the pairwise comparison of B18 vs. S18 fiber color formation. Highly expressed gene pathways included metabolic pathways (Ko01100, 709 genes), biosynthesis of secondary metabolites (ko01110, 431 genes), phenylpropanoid biosynthesis (ko00940, 73 genes), phenylalanine metabolism (ko00360, 14 genes), flavonoid biosynthesis (ko00941, 57 genes), flavone, and flavonol biosynthesis (ko00944, 14 genes) were identified (Figure 5A, Supplementary Materials). In B18 vs. Q18 pairwise comparison, 134 KEGG pathways were identified, in which metabolic pathways (Ko01100, 1139 genes), biosynthesis of secondary metabolites (ko01110, 600 genes), phenylpropanoid biosynthesis (ko00940, 126 genes), phenylalanine metabolism (ko00360, 23 genes), and flavonoid biosynthesis (ko00941, 68 genes) pathways were significantly induced (Figure 5B; Supplementary Materials). Moreover, as shown in Figure 5C, 134 pathways were enriched. Similarly, metabolic pathways (Ko01100, 527 genes), biosynthesis of secondary metabolites (ko01110, 289 genes), phenylpropanoid biosynthesis (ko00940, 80 genes), and flavonoid biosynthesis (ko00941, 28 genes) pathways were identified (Figure 5C; additional file 1). The top 50 GO terms in all three enriched categories were in DCF, LCF, and WCF at 18 DPA. As shown in Figure 5D, 37 GO terms were enriched in the category of biological process (e.g., plant type cell wall biogenesis, phenylpropanoid biosynthetic process, flavonoid metabolic process, flavonoid biosynthetic process), 2 GO terms in cell component (e.g., anchored component of plasma membrane), and 11 GO terms in molecular function (e.g., antiporter activity, glucosidase activity, transcription regulator recruiting activity). 

### 2.5. Co-Expression Network Analysis of Color-Related DEGs

**Figure 6:**
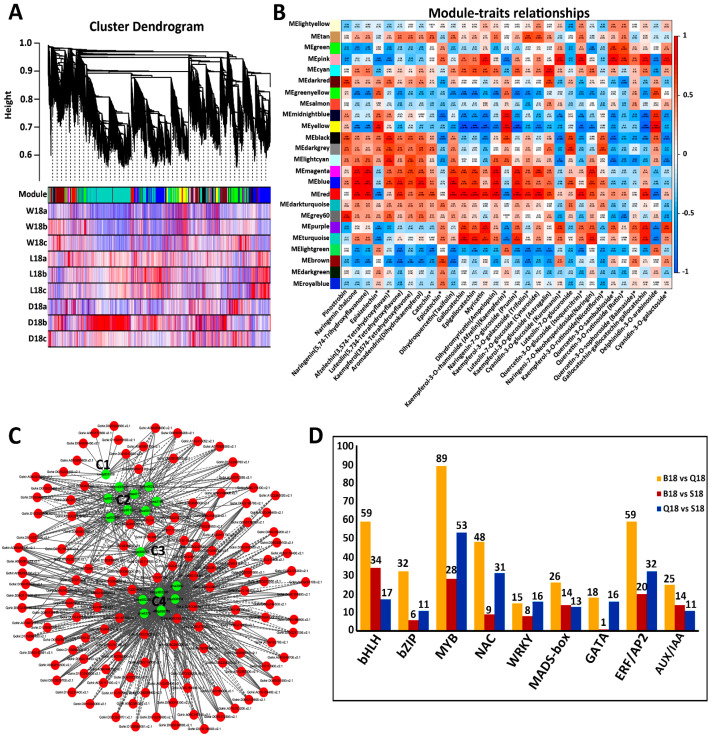
WGCNA of differential gene expressions and the related traits. (**A**) Hierarchical clustering dendrogram tree showing co-expression modules. Similar color-coded modules represent genes with similar expression patterns. (**B**) Module–trait relationship. The cells were assigned colors based on their statistical significance, labeled with two numbers; the lower number indicates the *p*-value and the upper number indicates the correlation coefficient. (*) denotes differentially expressed metabolites. (**C**) Correlation analysis between metabolites and genes. Metabolites are shown in green, and genes are shown in red, with solid lines representing positive correlations and dashed lines representing negative correlations. (**D**) The number of differentially expressed transcription factors.

To examine the gene regulatory network and specific gene modules during fiber pigment formation, 32,622 non-redundant DEGs were subjected to WGCNA. As shown in Figure 6A, WGCNA revealed major tress ranches with defined modules in which 24 distinct modules were labeled with different colors. Red, blue, and magenta-coded modules had the highest number of significant module–trait relationships. However, further results show that 6 module–trait relationships (blue-r = 0.92-naringenin chalcone, red-r = 0.94-epigallocatechin, magenta-r = 0.92-dihydomyricetin(ampelopsin), red-r = 0.94-naringenin-7-O-glucoside (prunin), yellow-r = 0.93-delpinidin-3-O-arabinoside, and pink-r = 0.95-quercetin-3-O-sambubioside) were highly significant (r >0.8, *p* <10−3) (Figure 6B). Furthermore, in order to find the relation between metabolite and gene hubs, correlation network analysis was conducted with a correlation coefficient of 0.88. A total of 16 metabolites, shown in green colors, and 141 genes hubs, shown in red colors, were annotated using cytoscape software (3.6.1 version). As shown in Figure 6C, four main metabolites-gene hubs clusters were formed. For instance, 1 metabolite at C1 and C3 formed a considerable amount of correlation with gene hubs. However, 8 metabolites at C2 and 6 metabolites at C4 formed a huge number of correlations (Figure 6C). Transcription factors play a role in anthocyanidin pigment formation [19]. Equally, in this study, a considerable number of transcription, namely (170) *MYB*, (49) *BZIP*, (110) *bHLH*, (53) *MADS*-box, (39) *WRKY*, (88) *NAC*, (111) *ERF/AP2*, and (50) *AUX/IAA* were significantly expressed in BCF, LCF, and WCF at 18 DPA (Figure 6D and Table S5).

### 2.6. Analysis and Verification of Structural Genes Involved Anthocyanin Biosynthesis Pathway

**Figure 7:**
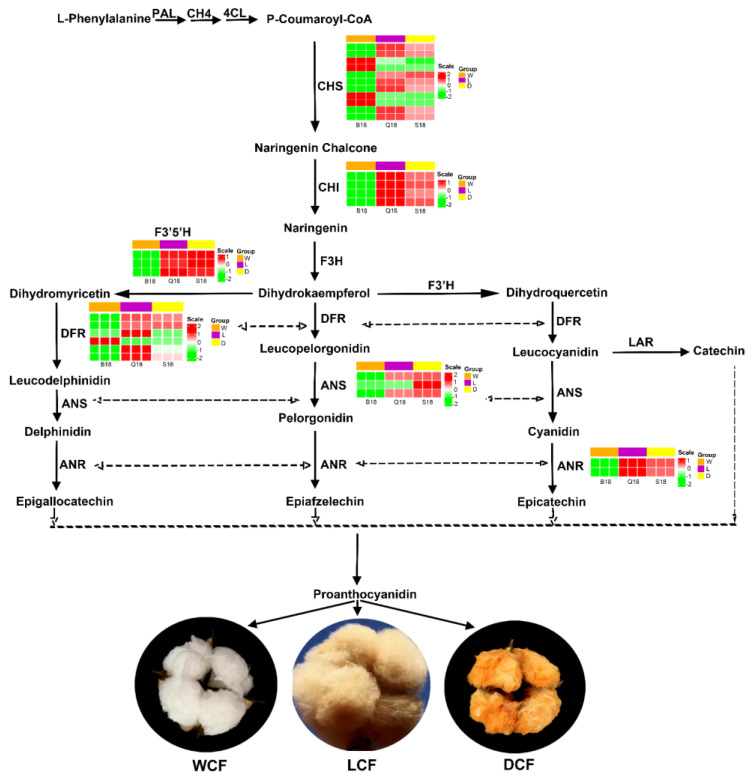
Schematic expression patterns of flavonoid-related genes at the different stages in WCF, LCF, and DCF. Enzyme abbreviations: PAL, phenylalanine ammonia lyase; C4H, cinnamate 4-hydroxylase; 4Cl, 4-coumarate: CoA ligase; CHS, chalcone synthase; CHI, chalcone isomerase; flavonoid 3′-hydroxylase; F3′5′H; DFR, dihydroflavonol 4-reductase; F3H, flavone 3-hydroxylase; F3′H, LAR, leucoanthocyanidin reductase; ANS, anthocyanidin synthase; ANR, anthocyanidin reductase; Heatmaps were created according to the average expression levels based on FPKM value. A color bar is presented on the right.

**Figure 8:**
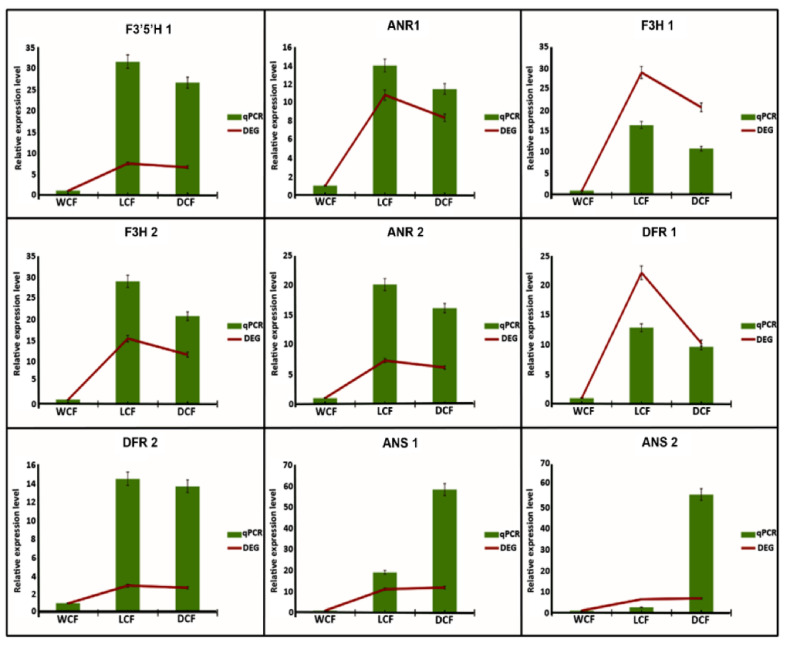
Validation of RNA-seq data by qRT-PCR. Columns represent the results of qRT-PCR, and zigzag lines indicate the results of differentially expressed gene levels.

According to KEGG pathway enrichment result, significantly expressed genes involved in flavonoid metabolic pathways were annotated using KOG, Swiss-Prot, eggnog, and NR database. Unigenes involved in the anthocyanin biosynthesis pathway were identified with the Log2Fc > 0.05. Anthocyanin belongs to a group of flavonoid family and a comprehensively studied plant secondary metabolism pathway popularly known to determine the color in plant organs [20]. Gene expressions in this pathway are grouped into two main categories comprising early genes: phenylalanine ammonialyase (*PAL*), cinnimate 4-hydroxylase (*C4H*), 4-coumarate: CoA ligase (*4CL*), chalcone synthase (*CHS*), Chalcone isomerase (*CHI*), and flavanone 3-hydroxylase (*F3H*), and late genes: dihydroflavonol reductase (*DFR*), anthocyanidin synthase (*ANS*) and UDPGlucose: flavonoid 3-Oglucosyltranferase *(UFGT*) [21]. As shown in Figure 7, genes of flavonoid biosynthesis were comprising chalcone synthase (*CHS*, 11 DEGs), dihydroflavonol reductase (*DFR*, 6 DEGs), chalcone isomerase (*CHI*, 4 DEGs), anthocyanidin synthase (*ANS*, 3 DEGs), anthocyanidin reductase (*ANR*, 2 DEGs), and flavonoid 3′,5′-hydroxylase (*F3′5′H*) in response to cotton fiber color formation at 18 DPA. In addition, most genes were down-regulated in WCF in comparison to DCF and LCF (Figure 7 and Table 1). At early structural gene expression of *CHS*, 7 genes (*Gohir.A05G403200.v2.1*, *Gohir.A05G318401.v2.1*, *Gohir.A10G121700.v2.1*, *Gohir.A10G121800.v2.1*, *Gohir.D02G031600.v2.1*, *Gohir.D10G144300.v2.1* and *Gohir.D10G144400.v2.1*) were down-regulated in WCF, but were up-regulated in DCF and LCF. On the contrary, 4 *CHS* genes (*Gohir.A09G000148.v2.1*, *Gohir.A09G000500.v2.1*, *Gohir.D09G000301.v2.1*, and *Gohir.D09G000401.v2.1*) were significantly up-regulated in WCF but were down-regulated in DCF and LCF (Figure 7 and Table 1). All four DEGs of *CHI* were up-regulated in both LCF and DCF with significant expression in LCF, but down-regulated in WCF at 18 DPA. Similar trends of gene expression were found in anthocyanidin synthase conversion of leucopelargonidin into pelargonidin. However, *F3′5′H* and *ANS* significant upregulation of DEGs were found in DCF compared to LCF (Figure 7 and Table 1). Moreover, late dihydroflavonol reductase structural genes were found to be more up-regulated in LCF compared to DCF but down-regulated in WCF (Figure 7). To further verify the expression profiling of significant DEGs involved in the anthocyanin pathway, 9 DEGs (i.e., *ANR1*, *ANR2*, *F3H1*, *F3H2*, *DFR1*, *DFR2*, *F3′5′H1*, *ANS1*, and *ANS2*) were subjected to quantitative real-time PCR. Primer sequences for each gene are listed in Table S6. All nine DEGs’ relative expressions were significantly lower in WCF (Figure 8). However, seven DEGs (*ANR1*, *ANR2*, *F3H1*, *F3H2*, *DFR1*, *DFR2*, and *F3′5′H1*) had the highest relative expressions in LCF (Figure 8). Moreover, two DEGs (*ANS1* and *ANS2*) activities were relatively high in DCF (Figure 8). We hypothesize that the high *ANR*, *F3H1*, *F3H2*, *ANS*, and *DFR* activity may explain the light brown coloration in LCF and DCF fiber coloration, while the relatively low activity may explain the white coloration in WCF. We speculate that our findings concerning qRT-PCR were suitable for illuminating the mechanisms of deep brown, light brown, and white cotton fiber coloration.

## 3. Discussion

### 3.1. Classifying Metabolites Differences of Pigment-Related in DCF, WCF and LCF

The molecular study of pigment formation in NBCC, with the aim of discovering stable brown-colored fibers and high fiber quality, is of great importance for developing cotton varieties suitable for textile industries. The identification of plant metabolites has been successfully established, ranging from 200,000 to 1 million species [22]. In contrast with other-omics, plant metabolome is thought to be closer to the phenotype of the organism capable of revealing the physiological phenomenon [23,24]. Cotton color pigmentation, using metabolome analysis, has been successfully investigated. For example, a comparative study of GCF revealed that caffeic acid and its derivatives positively correlate with the degree of green color in cotton fibers [8]. However, in brown cotton fibers, proanthocyanidins are thought to be the main components of the pigments deposited [12]. Condensed tannins are categorized as proanthocyanidins, with monomeric units of flavan-3-ols constituting a major part in brown cotton coloration [25,26]. Here, comparative analysis revealed a highly DEM tannin metabolite (i.e., Hexahydroxydiphenoyl acid-glucose) accumulated substantially in DCF and LCF in comparison with WCF (Tables S1 and S2). An existing study of hexahydroxydiphenoyls in cotton pigment formation is elusive; however, we speculate that high expression of the tannin metabolites may have a role to play in cotton brown color pigment formation. Procyanidins control pigment components in brown cotton fibers, but are mainly regulated by flavonoid pathway genes [27,28]. An analysis of flavonoid structural genes in brown fiber showed that the accumulation of naringenin, kaempferol, and myricetin in brown fiber significantly surpassed that in white cotton fiber at 12–21 DPA [16,28]. Consistent with our metabolome results, naringenin, kaempferol, and myricetin were found to be highly up-regulated in NBCC than WCF, however, there was significant expression in LCF at 18 DPA (Table S3). Recent studies have shown that PAs (flavan-3-ol units) are the main component of pigment deposition in BCF [28,29]. Xiao et al. further explain that most of flavan-3-ols (gallocatechin and catechin) in brown fiber are significantly up-regulated [11]. Similar to the present study, flavan-3-ol monomers, including afzelechin, epicatechin-epiafzelechin, and epigallocatechin, were up-regulated in LCF, while catechins were up-regulated in DCF. Interestingly, epiafzelechin metabolites were also found highly to be up-regulated in WCF (Figure 3D and Table S3). We speculate that up-regulation of flavone-3-ol monomers may influence cotton fiber and brown fiber development. Flavonoids are well-known to play a wide range of biological activities in plant UV protection, flower coloration, antioxidant, antibacterial, anti-inflammatories, anti-cancer and antifungal properties, and defense [27]. Contrary to brown color formation, PAs also have antioxidant and anti-inflammation activities [13]. NBCC biological activities are understudied. However, existing research has proven that NBCC have high flavonoid and phenolic pigment composition capable of antioxidant activities [7]. Luteolins are a type of flavonoid thought to possess anti-oxidant, anti-inflammatory, and anti-cancer features [30]. Similarly, chrysoeriol is a naturally occurring flavonoid known to possess antioxidant, antimicrobial, and anti-inflammatory characteristics. Chalcones play a key role in plant color formation but are also known to act as antibacterial agent [31,32]. Likewise, flanone-3-ols are popularly known to possess anti-inflammatory properties [29]. Consistent with the present study, flanone-3-ols, luteolin, chrysoeriol, and chalcone metabolites were highly up-regulated in LCF and DCF in comparison with WCF, however, higher expression was found in LCF (Figure 3A–E, Tables S1–S3), indicating that light brown cotton fiber may have high antioxidant, antimicrobial, and anti-inflammatory characteristics. It was hence hypothesized that the above-mentioned flavonoids may have high PA content. 

### 3.2. Genes Involved in Anthocyanidin Pathway Influence Fiber Pigment Development in NBBC 

Transcriptional analysis has revealed that the PA biosynthesis pathway wholly activates in brown cotton fiber. However, characterized flavonoid synthase genes, including *CHI*, *F3H*, *DFR*, *ANS*, *ANR*, *C4H*, *CHS*, and *F3′5H*, were highly expressed this pathway in BCF compared with their WCF counterparts. Many exiting studies have proven that the concentrations of PAs and its precursors in brown fiber were much higher than in white fiber [5,11,16]. Consistent with the present study, qRT-PCR analyses revealed that PA synthases, including *ANR*, *F3H*, *DFR*, *ANS*, and *ANR*, were significantly up-regulated in NBCC, indicating that the mentioned flavonoid synthase genes activate the accumulation of PA in NBCC (Table S1). Flavan-3-ols polymerization of monomeric composition is a key influencing factor of PA components [11]. Further genomic study of PA biosynthesis pathways revealed that two main branching points in the PA pathway lead to different PA monomers (Figure 7). First and foremost, DFR converts dihydrokampferol to leucoparlegonidin, leading to PA monomers with a single hydroxyl, then LAR converts leucoanthocyanidins to 2, 3-trans-flavan-3-ols (catechin and gallocatechin), while ANS catalyzes leucoanthocyanidins to form anthocyanindins (epicatechin and epigallocatechin) with ANR activity [11]. Hence, PAs were synthesized through anthocyanin biosynthesis pathway under the activities of LAR or ANS/ANR biosynthetic routes [33]. Anthocyandin transport is key to proanthocyandin synthesis. Therefore, silencing anthocyandidn genes such as *GhANR*, *GhLAR*, and *GhCHS* significantly interfered with colored cotton fiber with different color depth [15]. Recent reports show that flavonoid synthase genes, including *CHI*, *F3H*, *DFR*, *ANS*, and *ANR*, were significantly expressed in NBCC at 20 DPA, with few significant expressions in WCF [33]. Similar findings were also discovered by Li et al., indicating significant expression of *CHS*, *CHI*, and *ANR* in BCF compared to WCF [14]. Similar to the present study, *CHS*, *CHI*, *ANS*, *F3′5′H*, and *ANR* were up-regulated in NBCC with significant expression in LCF (Figure 7, Table 1). We hypothesized that brown fiber inter- and intra-pigment differences in this study may be regulated by the *ANS/ANS* expression route. Xiao et al. argued that brown cotton fibers contain primarily catechin and gallocatechin, suggesting that proanthocyanidins are synthesized via the *LAR* route rather than the *ANS/ANR* routes [11]. However, in some cases, some flows through *ANS* route, resulting in anthocyanidins converted by *ANR* into PAs. 

Transcription factors play a significant role in plant pigment formation. Transcription factors such as *IAA/AUX* [34], *ARF* [35], *MYB* [36,37], *bHLH* [38], and *WRKY* [20] have been reported to regulate anthocyanidin accumulation and pigment formation. Here, similar transcription factors were expressed (Figure 6 and Table S5). In relation to flavonoid biosynthesis and anthocyanidin accumulation, transcription factors such as *R2R3-MYB* domain, a *bHLH* (basic helix-loop-helix) domain, and conserved WD40 repeats (*WDRs*) have been reported to be key regulators [39]. Consistent with previously reported studies, over-expressing of grapevine *VvMYB5a* and *VvMYB5b* produced changes in flavonoid, chlorophyll, and β-carotene production [40]. In Petunia, floral anthocyanin pigmentation patterns expression patterns were reported to be determined by *R2R3-MYB* and *bHLH* activators. In addition, *R2R3-MYB* proteins activated early anthocyanins and PA biosynthetic genes [13]. Moreover, some reports have confirmed that *MYB* encoding *TT2* homolog gene *GhTT2-3A* controls PA biosynthesis and dark brown pigmentation in cotton fiber coloration, and high-level expression of *GhTT2_A07* results in the up-regulation of the PA pathway [17]. In this study, *MYB* and *bHLH* transcription factors were found to be significantly expressed in NBCC (Figure 6D and Table S5). In addition, *MYB*-encoding transcription factor *TT2*-like genes were significantly up-regulated in BCF compared to LCF (Table S5). Hence, we hypothesized that transcription factors *MYB* and *BHLH* may play significant roles in brown color pigmentation and likely the expression of PA-related genes. However, molecular and cellular analysis regarding *MYB* in relation to BCF and LCF formation requires further elucidation. In summary, our results have proven that higher brown color intensity may not guarantee high anticyanidin, PA, and fiber color durability. The result clearly shows that endogenous genes expressions in LCF are significantly higher than in DCF and WCF. These results provide novel insights into the molecular regulation of anthocyanidin biosynthesis and accumulation in the process of fiber color change in cotton. Thus, these results may facilitate genetic modification or selection for further improvement in the color of DCF.

## 4. Materials and Methods 

### 4.1. Plant Materials

White cotton Simian 3 and two brown cotton lines were used in this study, and all accessions belonging to *G. hirsutum* were planted in the experimental field of Anhui Agricultural University. Conventional practices of cultivation were used throughout the growth period. Samples were collected from developing bolls at 18 days post-anthesis (DPA), and were replicated three times. All samples were collected from 7:00–10:00 a.m. to minimize potential variability associated with circadian rhythms. Samples were frozen immediately in liquid nitrogen and stored at −80◦C until use.

### 4.2. RNA Extraction and RNA-Seq Analysis

Total RNA was extracted from 1.0 g of cotton fiber of DCF, LCF and WCF using RNA Purification Kit according to the manufacturer’s protocol (Sangon Biotech, Shanghai, China). The RNA-seq sequencing and assembly were conducted by the MetWare Technologies Corporation (Wuhan, China). The Agilent 2100 BioAnalyzer was used to detect the fragment size and concentration of the library. The combined probe anchoring polymerization (CPAS) technique was used for sequencing, and the read length of 150 bp was obtained. The library was constructed and sequenced on the Illumina HiSeq 2500 platform [41]. Following the removal of low-quality sequence reads, clean reads were mapped to the reference genome sequence (https://www.cottongen.org (accessed on 14 January 2021)) using HISAT2 program.

### 4.3. Functional Annotation and Expression Level Analysis

The DESeq R package was used to identify differentially expressed genes (DEGs) in each sample group based on a *p*-value of <0.05 and a fold change of ≥2.0. The following databases were used for gene function annotation and pathway analysis: NR (NCBI non-redundant protein sequence), NT (NCBI non-redundant nucleotide sequence), Pfam (protein family), KOG/COG (Clusters of Orthologous Groups of proteins), Swiss-Prot (manually annotated and commented protein sequence), KEGG (Kyoto Encyclopedia of Genes and Genomes), KO (KEGG Ortholog), and GO (Gene Ontology). Gene expression levels were estimated by the FPKM (fragments per kilobase of transcript per million fragments mapped) method.

### 4.4. Weighted Gene Co-Expression Network Analysis (WGCNA)

WGCNA analysis was performed on the transcriptome data to identify highly coordinated gene sets during fruit development in green and red prickly ash, as well as associations between gene sets and aroma. Genes with FPKM > 1 were used to construct a weighted gene co-expression network and partition module with the WGCNA v1.6.6 package in R v3.4.4. The block wise Modules function was used to build a scale-free network, and all parameters were set to default values [42]. 

### 4.5. Sample Preparation, Metabolite Extraction, and UPLC-ESI-MS/MS Analysis 

Biological samples were freeze-dried by a vacuum freeze-dryer (Scientz-100F). The freeze-dried sample was crushed using a mixer mill (MM 400, Retsch, Haan, Germany) with a zirconia bead for 1.5 min at 30 Hz. We dissolved 100 mg of lyophilized powder with 1.2 ml 70% methanol solution, vortexed for 30 s every 30 min for 6 times in total, and placed the sample in a refrigerator at 4 °C overnight. Following centrifugation at 12,000 rpm for 10 min, the extracts were filtrated (SCAA-104, 0.22 μm pore size; ANPEL, Shanghai, China, http://www.anpel.com.cn/ (accessed on 14 January 2021)) before UPLC-MS/MS analysis. The sample extracts were analyzed using an UPLC-ESI-MS/MS system (UPLC, SHIMADZU Nexera X2, www.shimadzu.com.cn/; MS, Applied Biosystems 4500 Q TRAP, www.appliedbiosystems.com.cn/). The analytical conditions were as follows. UPLC: column, Agilent SB-C18 (1.8 µm, 2.1 mm × 100 mm). The mobile phase consisted of solvent A, pure water with 0.1% formic acid, and solvent B, acetonitrile with 0.1% formic acid. Sample measurements were performed with a gradient program that employed the starting conditions of 95% A and 5 % B. Within 9 min, a linear gradient to 5% A, 95% B was programmed, and a composition of 5% A, 95% B was kept for 1 min. Subsequently, a composition of 95% A,5.0 % B was adjusted within 1.10 min and kept for 2.9 min. The flow velocity was set as 0.35 mL per minute; The column oven was set to 40 °C. The injection volume was 4μL. The effluent was alternatively connected to an ESI-triple quadrupole-linear ion trap (QTRAP)-MS.

### 4.6. ESI-Q TRAP-MS/MS

LIT and triple quadrupole (QQQ) scans were acquired on a triple quadrupole-linear ion trap mass spectrometer (Q TRAP), AB4500 Q TRAP UPLC/MS/MS System, equipped with an ESI Turbo Ion-Spray interface, operating in positive and negative ion mode, and controlled by Analyst 1.6.3 software (AB Sciex, Framingham, MA, USA). The ESI source operation parameters were as follows: ion source, turbo spray; source temperature 550 °C, ion spray voltage (IS) 5500 V (positive ion mode)/−4500 V (negative ion mode), ion source gas I (GSI), gas II (GSII), and curtain gas (CUR) were set at 50, 60, and 25.0 psi, respectively; the collision-activated dissociation (CAD) was high. Instrument tuning and mass calibration were performed with 10 and 100 μmol/L polypropylene glycol solutions in QQQ and LIT modes, respectively. QQQ scans were acquired as MRM experiments with collision gas (nitrogen) set to medium. DP and CE for individual MRM transitions were done with further DP and CE optimization. A specific set of MRM transitions were monitored for each period according to the metabolites eluted within this period.

### 4.7. Selection of Differential Metabolites 

Significantly regulated metabolites between groups were determined by VIP ≥ 1 and absolute Log2FC (fold change) ≥ 1. VIP values were extracted from the OPLS-DA result, which also contains score plots and permutation plots, and was generated using R package MetaboAnalystR. The data was log transformed (log2) and mean-centered before OPLS-DA. In order to avoid overfitting, a permutation test (200 permutations) was performed.

### 4.8. qRT-PCR Validation 

To validate the results of RNA-seq, quantitative real-time PCR (qRT-PCR) was performed. Nine DEGs (*ANR1*, *ANR2*, *F3H1*, *F3H2*, *DFR1*, *DFR2*, *F3′5′H1*, *ANS1*, and *ANS2*) were selected for qRT-PCR analysis to verify the expression patterns in DCF, LCF, and WCF. The respective qRT-PCR primers are listed in Table S5. Total RNA was extracted from upland cotton fiber using TRIzol Reagent. cDNA synthesis was performed using an RT reagent kit (Tiangen, Beijing, China). qRT-PCR was analyzed in a 20 μL reaction system (including 10 μL SYBR Premix Ex Taq ™ Ⅱ (2×), 2 μL cDNA, and 0.8 μL upstream and downstream primers) and a simple procedure (50 °C for 2 min; 40 cycles at 95 °C for 30 s, 95 °C for 5 s, and 60 °C for 20 s, and a final extension at 72 °C for 10 min). The GhUBQ gene was used as a control, and each sample was repeated 3 times. The relative expression levels were calculated using the 2^−ΔΔCt^ method [43]. 

## Figures and Tables

**Table 1:** List of the flavonoid biosynthesis pathway genes.

Gene ID	Symbol	DCF vs. LCF	DCF vs. WCF	LCF vs. WCF	Description
		Log_2_FC	Log_2_FC	Log_2_FC	
*Gohir.A05G167300.v2.1*	*ANR*	2.88	2.62	−0.24	Anthocyanidin reductase
*Gohir.D05G170300.v2.1*	*ANR*	3.44	3.07	−0.36	
*Gohir.A08G176400.v2.1*	*LDOX/ANS*	3.48	3.57	0.11	Leucoanthocyanidin dioxygenase
*Gohir.D04G067900.v2.1*	*LDOX/ANS*	0.12	1.63	1.51	
*Gohir.D08G195100.v2.1*	*LDOX/ANS*	2.67	2.77	0.11	
*Gohir.A05G403200.v2.1*	*CHI*	2.15	1.86	−0.28	Chalcone isomerase
*Gohir.A13G020400.v2.1*	*CHI*	2.42	2.21	−0.19	
*Gohir.D04G012300.v2.1*	*CHI*	2.29	1.87	−0.41	
*Gohir.D13G021000.v2.1*	*CHI*	1.67	1.45	−0.21	
*Gohir.A02G024200.v2.1*	*CHS*	3.16	2.80	−0.36	Chalcone synthase
*Gohir.A05G318401.v2.1*	*CHS*	2.72	2.52	−0.20	
*Gohir.A09G000148.v2.1*	*CHS*	−1.00	−1.55	−0.53	
*Gohir.A09G000500.v2.1*	*CHS*	−1.79	−1.61	0.18	
*Gohir.A10G121700.v2.1*	*CHS*	4.75	4.92	0.17	
*Gohir.A10G121800.v2.1*	*CHS*	3.61	3.37	−0.22	
*Gohir.D02G031600.v2.1*	*CHS*	2.96	2.60	−0.35	
*Gohir.D09G000301.v2.1*	*CHS*	−1.30	−1.40	−0.09	
*Gohir.D09G000401.v2.1*	*CHS*	−1.58	−1.61	−0.02	
*Gohir.D10G144300.v2.1*	*CHS*	3.07	2.70	−0.36	
*Gohir.D10G144400.v2.1*	*CHS*	3.17	2.87	−0.29	
*Gohir.A05G192500.v2.1*	*DFR*	1.55	1.43	−0.11	Dihydroflavonol 4-reductase
*Gohir.A06G008700.v2.1*	*DFR*	2.83	2.81	−0.02	
*Gohir.A07G138900.v2.1*	*DFR*	4.20	0.27	−3.86	
*Gohir.D01G059900.v2.1*	*DFR*	−1.46	−1.21	0.27	
*Gohir.D05G195700.v2.1*	*DFR*	4.47	3.36	−1.10	
*Gohir.D06G004300.v2.1*	*DFR*	2.98	2.36	−0.61	
*Gohir.A07G120800.v2.1*	*F3′5′H*	3.14	3.24	0.11	Flavonoid 3′,5′-hydroxylase
*Gohir.A07G120900.v2.1*	*F3′5′H*	2.78	2.98	0.21	
*Gohir.D07G125100.v2.1*	*F3′5′H*	2.90	2.71	−0.18	

## Supplementary Materials

The following supporting information can be downloaded at: https://www.mdpi.com/article/10.3390/ijms24065249/s1.

## Abbreviations

NBCC	Naturally brown cotton
DCF	dark brown cotton fiber
LCF	light brown cotton fiber
WCF	white cotton fiber
DEGs	differentially expressed genes
DeMs	differentially expressed metabolites
PA	Proanthocyanidin
DPA	days post-anthesis
CHS	chalcone isomerase
F3H	flavanone 3-hydroxylase
F3′H	flavanone 3-hydroxylase
DFR	dihydroflavonol 4-reductase
F3′5′H	flavanone-3′5′-hydroxylase
ANS	anthocyanindin synthase
CHI	chalcone isomerase
