# Supplementary material for: Transcriptome and Metabolome Profiling Unveil Pigment Formation Variations in Brown Cotton Lines (Gossypium hirsutum L.)
Source: Int J Mol Sci. 2023 Mar 9;24(6):5249. doi: 10.3390/ijms24065249 (PMC10049672; doi:10.3390/ijms24065249)
Supplement: Supplementary file 1 [file ijms-24-05249-s001.zip › ijms-2233301-supplementary.pdf]

Table S1. Top 20 differentially expressed metabolites in W18 vs L18 comparison

Index	Formula	Metabolite Name	Log2 (Fold Change) WCF/LCF	Class	VIP
lmhn000921	$C_{20}H_{18}O_{14}$	3, 4, 5, 3', 4', 5'-Hexahydroxydiphenoyl acid-glucose	16.33	Tanin	1.38
mws1639	$C_{11}H_{10}O_5$	Isofraxidin	13.93	Coumarin	1.34
mws0180	$C_7H_6O_4$	2,5-Dihydroxybenzoic acid; Gentisic Acid	13.52	Phenolic acid	1.38
pmb3012	$C_{22}H_{22}O_{11}$	Chrysoeriol-7-O-glucoside*	13.41	Flavonoid	1.38
hmpn005101	$C_{21}H_{24}O_{11}$	Sieboldin	13.36	Chalcone	1.39
cmxn004016	$C_{21}H_{24}O_{11}$	Dihydromarein	13.25	Chalcone	1.38
pmb2999	$C_{22}H_{22}O_{11}$	Chrysoeriol-5-O-glucoside*	13.17	Flavonoid	1.38
pmb3062	$C_{17}H_{22}O_{10}$	1-O-Eudesmoylquinic acid	12.05	Phenolic acid	1.38
lmbn014696	$C_{20}H_{30}O_2$	Pimaric acid	11.98	Ditepenoid	1.38
Pmb3000	$C_{24}H_{24}O_{12}$	Chrysoeriol-7-O-(6''-acetyl)glucoside	11.98	Flavonoid	1.38
zmxp003107	$C_{27}H_{30}O_{16}$	Luteolin-7,3'-di-O-glucoside*	-15.51	Flavonoid	1.38
hmbp002498	$C_{21}H_{26}O_{13}$	Scopoletin-7-O-xylosyl(1→6)glucoside	-13.44	Coumarin	1.38
hmpp002612	$C_{27}H_{30}O_{16}$	Luteolin-7-O-gentiobioside*	-13.19	Flavonoid	1.38
zmxp002867	$C_{28}H_{32}O_{16}$	Chrysoeriol-5,7-di-O-glucoside	-9.34	Flavonoid	1.37
hmqp002870	$C_{32}H_{38}O_{20}$	Luteolin-7-O-Sophoroside-5-O-arabinoside	-4.17	Flavonoid	1.37
lmhn002573	$C_{17}H_{20}O_{11}$	Sinapoylglucuronic acid	-3.70	Phenolic acid	1.37
lmgn002253	$C_{31}H_{36}O_{18}$	Syringoylcaffeoylquinic acid-D-glucose	-3.57	Phenolic acid	1.32
hmcn002743	$C_{22}H_{26}O_8$	Lirioresinol A	-3.03	Lignan	1.00
hmtp000776	$C_8H_9NO_3$	4,5,6-Trihydroxy-2-cyclohexen-1-ylideneacetonitrile	-2.81	Alkaloid	1.38
pmb4777	$C_{16}H_{18}O_8$	4-Hydroxy-7-methoxycoumarin-β-rhamnoside*	-2.51	Coumarin	1.35

Table S2. List of top 20 differentially expressed metabolites in W18 vs D18

Index	Formula	Metabolite Name	Log2 (Fold Change) WCF/DCF	Class	VIP
lmhn000921	$C_{20}H_{18}O_{14}$	3, 4, 5, 3', 4', 5'-Hexahydroxydiphenoyl acid-glucose	16.04	Tanin	1.38
cmxn004016	$C_{21}H_{24}O_{11}$	Dihydromarein	13.72	Chalcone	1.38
lmmp003349	$C_{19}H_{30}O_{7}$	Byzantionoside A	13.69	Others	1.38
hmpn005101	$C_{21}H_{24}O_{11}$	Sieboldin	13.61	Chalcone	1.39
lmzn006284	$C_{30}H_{48}O_{4}$	2α-Hydroxyursolic acid	13.61	Triterpene	1.36
mws0180	$C_{7}H_{6}O_{4}$	2,5-Dihydroxybenzoic acid; Gentisic Acid	13.14	Phenolic acid	1.38
pmb3012	$C_{22}H_{22}O_{11}$	Chrysoeriol-7-O-glucoside	12.63	Flavonoid	1.38
pmb2999	$C_{22}H_{22}O_{11}$	Chrysoeriol-5-O-glucoside	12.59	Flavonoid	1.38
pmb3062	$C_{17}H_{22}O_{10}$	1-O-Eudesmoylquinic acid	12.14	Phenolic acid	1.38
pmn001468	$C_{19}H_{26}O_{13}$	Sibiricose A3	11.44	Phenolic acid	1.38
zmxp003107	$C_{27}H_{30}O_{16}$	Luteolin-7,3'-di-O-glucoside	-15.51	Flavonoid	1.38
hmbp002498	$C_{21}H_{26}O_{13}$	Scopoletin-7-O-xylosyl(1→6)glucoside	-13.44	Coumarin	1.38
hmqp003066	$C_{35}H_{40}O_{18}$	Luteolin-7-O-(6'''-malonyl)sophoroside-5-O-arabinoside	-12.41	Flavonoid	1.38
mws1175	$C_{16}H_{14}O_{4}$	Pinostrobin	-9.84	Flavonoid	1.36
mws4167	$C_{21}H_{18}O_{12}$	Luteolin-7-O-glucuronide	-9.79	Flavonoid	1.37
pmn001702	$C_{21}H_{18}O_{12}$	Tetahydroxyflavone-7-O-glucuronide	-9.70	Flavonoid	1.37
lmhn002573	$C_{17}H_{20}O_{11}$	Sinapoylglucuronic acid	-7.92	Phenolic acid	1.37
pmb4777	$C_{16}H_{18}O_{8}$	4-Hydroxy-7-methoxycoumarin-β-rhamnoside	-5.24	Coumarin	1.35
hmqp002870	$C_{32}H_{38}O_{20}$	Luteolin-7-O-Sophoroside-5-O-arabinoside	-4.90	Flavonoid	1.37
lmgn002253	$C_{31}H_{36}O_{18}$	Syringoylcaffeoylquinic acid-D-glucose	-4.06	Phenolic acid	1.36

Table S3. List of top 20 differentially expressed metabolites in L18 vs D18 comparison

Index	Formula	Metabolite Name	Log_2 (Fold Change) LCF/DCF	Class	VIP
lmzn006284	$C_{30}H_{48}O_4$	2α-Hydroxyursolic acid	13.61	Triterpene	1.50
pmn001369	$C_{32}H_{42}O_{17}$	1-Hydroxypineolin Diglucoside	9.51	Lignans	1.52
zmxp002867	$C_{28}H_{32}O_{16}$	Chrysoeriol-5,7-di-O-glucoside	8.81	Flavonoid	1.37
pmp001005	$C_{15}H_{12}O_5$	1-Hydroxy-3,7-dimethoxyxanthone	4.54	Xanthone	1.42
lmmn005958	$C_{15}H_{16}O_3$	3-Hydroxy-furocalamen-2-one	4.47	Others	1.15
pmn001415	$C_{24}H_{20}O_9$	Catechin-(7,8-bc)-4β-(3,4-dihydroxyphenyl)-dihydro-2-(3H)-one	3.65	Flavanol	1.39
pmn001416	$C_{24}H_{20}O_9$	Catechin-(7,8-bc)-4α-(3,4-dihydroxyphenyl)-dihydro-2-(3H)-one*	3.58	Flavanol	1.37
hmgp002618	$C_{21}H_{22}O_{10}$	Isosalipurposide (Phlorizin Chalcone)	3.19	Chalcone	1.43
pmp000589	$C_{24}H_{22}O_{15}$	Quercetin-7-O-(6''-malonyl)glucoside	3.03	Flavonol	1.30
hmln002189	$C_{24}H_{22}O_{15}$	Quercetin-3-O-(6''-malonyl)galactoside	2.86	Flavonol	1.41
pmn001702	$C_{21}H_{18}O_{12}$	Tetahydroxyflavone-7-O-glucuronide	-11.99	Flavonoid	1.46
mws4167	$C_{21}H_{18}O_{12}$	Luteolin-7-O-glucuronide	-11.81	Flavonoid	1.50
mws1175	$C_{16}H_{14}O_4$	Pinostrobin	-10.19	Flavonoid	1.36
hmqp003066	$C_{35}H_{40}O_{18}$	Luteolin-7-O-(6'''-malonyl)sophoroside-5-O-arabinoside	-12.41	Flavonoid	1.38
lmhn002573	$C_{17}H_{20}O_{11}$	Sinapoylglucuronic acid	-4.21	Phenolic acid	1.50
mws2367	$C_{14}H_{20}O_7$	Salidroside	-3.64	Phenolic acid	1.50
lmtn002916	$C_{22}H_{20}O_{14}$	Mearnsetin-3-O-glucuronide	-3.29	Flavonoid	1.19
pme3285	$C_{15}H_{14}O_5$	Afzelechin (3,5,7,4'-Tetrahydroxyflavan)	-3.24	Flavanol	1.41
lmmp002529	$C_{33}H_{40}O_{21}$	Gossypetin-3-O-rutinoside-8-O-rhamnoside	-3.06	Flavonol	1.18
lmhn003074	$C_{14}H_{14}O_8$	Feruloylmalic acid	-2.88	Phenolic acid	1.50

Table S4. Transcriptome sequencing quality statistics of cotton fiber

Sample	Raw Reads	Clean Reads	Clean Base(G)	Error Rate(%)	Q20	Q30	GC Content(%)
B18a	506,012,90	48,679,844	7.3	0.02	98.19	94.5	44.39
B18b	566,570,16	54,601,176	8.2	0.02	98.35	94.92	44.12
B18c	590,625,96	56,644,984	8.5	0.02	98.32	94.81	44.17
Q18a	554,028,02	51,282,678	7.7	0.02	98.43	95.09	44.09
Q18b	580,525,30	55,213,036	8.3	0.02	98.43	95.06	44.11
Q18c	652,920,36	61,998,236	9.3	0.02	98.3	94.71	44.24
S18a	502,940,62	47,496,038	7.1	0.03	97.64	93.09	44.34
S18b	539,224,72	51,689,246	7.8	0.02	98.3	94.74	43.86
S18c	511,504,78	47,514,852	7.1	0.02	98.41	95.07	44.07

Tables S5. List of differentially expressed transcription factor in DCF, LCF and WCF at 18 DPA

Gene ID	TF Family	B18 vs Q18	B18 vs S18	Q18 vs S18	Description
		Log2 FC	Log2FC	Log2FC	
Gohir.A05G074600.v2.1	*B3-ARF*	2.93	ND	-2.97	Auxin response factor 4
Gohir.A05G187900.v2.1	*B3-ARF*	-1.94	-3.87	ND	Auxin response factor 5
Gohir.D06G193900.v2.1	*AUX*	3.79	ND	-4.56	Auxin-induced protein AUX22
Gohir.A08G111000.v2.1	*MYB*	4.72	3.76	ND	Transcription factor *MYB111*
Gohir.A07G020200.v2.1	*MYB*	6.50	6.31	ND	Myb-related protein 123
Gohir.D06G074700.v2.1	*MYB*	4.02	2.92	ND	Transcription factor *MYB3*
Gohir.A13G099200.v2.1	*MYB*	2.28	1.83	ND	Myb-related protein 5
Gohir.A06G075700.v2.1	*MYB*	2.70	2.12	ND	Transcription factor *TT2*-like
Gohir.D06G074700.v2.1	*MYB*	4.02	2.92	ND	Transcription factor *TT2*-like
Gohir.A08G182800.v2.1	*bHLH*	2.94	ND	ND	Transcription factor *bHLH1*
Gohir.D10G208450.v2.1	*NAC*	-1.07	ND	ND	NAC domain containing protein 52
Gohir.A13G064566.v2.1	*NAC*	-3.19	ND	ND	NAC domain-containing protein 19-like
Gohir.D06G189700.v2.1	*WRKY*	1.31	ND	-1.43	Probable *WRKY* transcription factor 40
Gohir.D11G113700.v2.1	*WRKY*	2.33	ND	-3.25	WRKY transcription factor 22
Gohir.D01G045300.v2.1	*bZIP*	2.57	1.25	-1.31	Basic leucine zipper 61

ND denotes not detected expression

Table S6. The primers used for qRT-PCR

	Gene ID	Forward Primer sequences (3′-5′)	Reverse Primer sequences (5′-3′)
F3′5′H1	*Gohir.A07G120900.v2.1*	CGAGGAGATGGATAAGGTGATTGGC	GGTGGACACTCTAGGCAAGTTCAAG
ANR1	*Gohir.D05G170300.v2.1*	GCCAGCCAGATCGTAGGAACAAAG	GAGCAACAACTTGACCAGCAATGAC
ANR2	*Gohir.A05G167300.v2.1*	CCAGCCAGCTCGTAGGAACAAAG	GCAACAACTTGACCAGCAATGAGG
F3H1	*Gohir.D12G059800.v2.1*	CTCCTTCAACTCTGACGGCTCTTG	CCTTAGGACGCTCATCTTCATCACG
F3H2	*Gohir.A12G059400.v2.1*	ATTGTTGAGGCTTGTGAGGATTGGG	CTCTCTCGCAAGACGGGTCATTTC
DFR1	*Gohir.D05G195700.v2.1*	CGCCTCTCACCATGCTACCATTATC	CCACACTCTTCAGGTTCTCATCCAC
DFR2	*Gohir.A05G192500.v2.1*	CGCCTCTCACCATGCTACCATTATC	CCACACTCTTCAGGTTCTCATCCAC
ANS1	*Gohir.D08G195100.v2.1*	GGAAGTTGGTGGCGTGGAAGAG	GAGCGAGTTCTGGTTGTGGACAC
ANS2	*Gohir.A08G176400.v2.1*	GGAAGTTGGTGGCGTGGAAGAG	GAGCGAGTTCTGGTTGTGGACAC

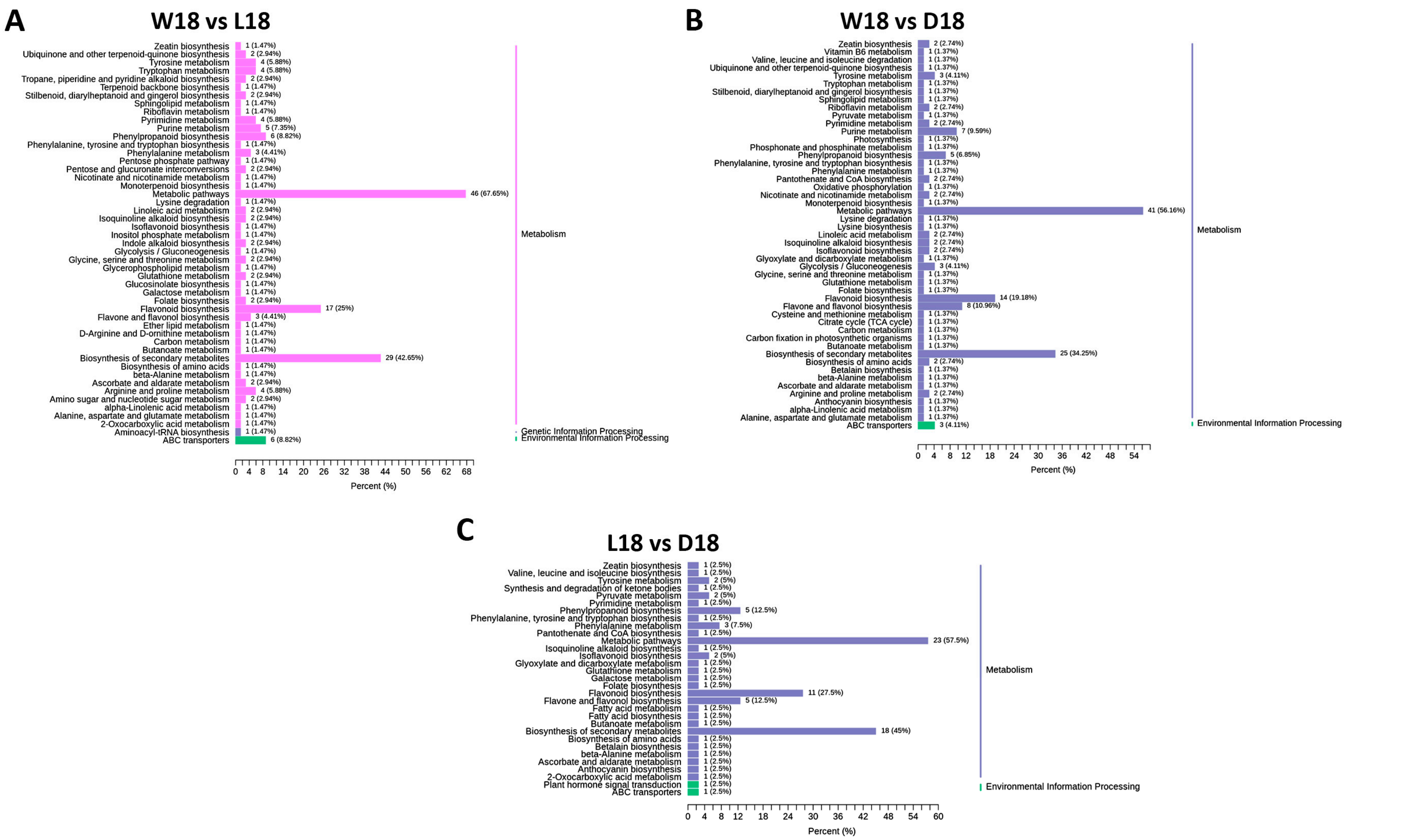

Figure S1. Kyoto Encyclopedia of Genes and Genomes (KEGG) pathway analysis of metabolites. The ordinate represents the KEGG metabolic pathway, and the abscissa, the number of metabolites annotated to the pathway and the proportion of the total number of metabolites annotated. L18, D18 and W18 represent deep brown fiber, light brown fiber and white fiber at 18 DPA.
